# Randomized controlled trial protocol: balance training with rhythmical cues to improve and maintain balance control in Parkinson’s disease

**DOI:** 10.1186/s12883-015-0418-x

**Published:** 2015-09-07

**Authors:** Tamine Teixeira da Costa Capato, Juliana Tornai, Patrícia Ávila, Egberto Reis Barbosa, Maria Elisa Pimentel Piemonte

**Affiliations:** Department of Physicaltherapy, University of São Paulo, Av Dr Enéias de Aguiar, 255 – 05403.000 São Paulo, São Paulo Brazi; PHYSICAL, Rua Cubatão 929 conj, 142 - 04013-043 São Paulo, São Paulo Brazil; Department of Neurology, University of São Paulo, Av Dr Enéias de Aguiar, 255 – 05403.000 São Paulo, São Paulo Brazil

**Keywords:** Randomized clinical trial, Parkinson’s disease, Physical therapy, Balance training, Postural control, Cues

## Abstract

**Background:**

Postural instability is a particularly incapacitating disorder, whose loss of motor independence by Parkinson´s Disease (PD) patients marks a significant stage of disease onset. Evidence suggests that deficits in automatic motor control, sensory integration and attention are associated with the lack of balance in PD. Physiotherapy together with medication play an important role in the treatment of this state, although no consensus has been reached on the best treatment modality. The aim of this randomized controlled trial protocol is to evaluate the effects of balance training with rhythmical (BRT), which is a motor program to improve balance associated with rhythmical auditory cues (RACs). This study is ongoing in the stage 1.

**Methods and design:**

A total of 150 PD patients at H&Y stages II–III and asymptomatic for depression and dementia are enrolled in a single-blind randomized study. Randomization is achieved via a computer-generated random-sequence table. All patients should also present a fall history. They will be assigned into one of three groups, and their balance and gait will be assessed before and after 10 training sessions, and after 4 and 30 weeks subsequent to the end of the training. The BRT group will receive a motor program to improve balance associated with RACs, the MT group will perform motor training with the same aims as those in the BRT group but without RACs, and the control group (CG) will be trained only in orientations. The exercise program specific to balance is of 5 weeks’ duration with two sessions per week, 45 min each, and consists of general physiotherapy exercises. Each session will be divided into five warm-up minutes—30 min for the main part and 10 min for the cool down. The training progresses and intensifies each week depending on the individual’s performance. The subjects should be able to execute 10 repetitions of the exercise sequences correctly to progress to the next movement.

**Discussion:**

This randomized study protocol will evaluate the effects of a motor program designed to improve balance associated with RACs, and will also assess whether balance training leads to activation of balance reactions at the appropriate time. We hypothesize that if this motor program is maintained long-term, it will prevent falls.

**Trial registration:**

Clinicaltrials.gov NCT02488265; Ethics Committee of the University of São Paulo Faculty of Medicine Clinics Hospital 1.102.464.

**Electronic supplementary material:**

The online version of this article (doi:10.1186/s12883-015-0418-x) contains supplementary material, which is available to authorized users.

## Background

The balance disturbances of Parkinson’s disease (PD) are progressive and limit the functional independence of the patients, thus affecting their quality of life [1]. The balance disturbance and freezing correlate with the evolution and severity of the PD and can be presented- in other parkinsonian syndromes. These symptoms are more apparent and debilitating when patients show midline signs and cognitive declines (especially those of executive function) [2].

The neural control of posture is compromised in patients with PD [3, 4]. Automatic postural responses can be influenced by cortical processing related to learning, previous experiences, and initial postural conditions [5]. The basal nucleus plays an important role in the control of axial tone [6, 7] and postural responses [8] and in the interpretation of somatosensory information [9]. Nevertheless, the dopaminergic loss in the basal ganglia not only affects automaticity, but also causes cognitive decline in executive functions, especially mental flexibility and set-shifting related to alterations in fronto-striatal connectivity [10]. The execution of challenging voluntary tasks leads to reduced postural stability in individuals with PD, whereas no influence on the voluntary task performance is observed [11, 12]. Currently available techniques have not been adopted yet by practitioners in the treatment of routine clinical assessment gait and balance disturbances [13].

Physiotherapy is commonly prescribed in association with medical and surgical treatments [14], aiming to reduce functional losses due to motor alterations in PD [15]. Recently, there have been strong evidences that exercise can improve motor [16–18] and cognitive performance [19] in PD. External sensory cues (auditory, visual and somatosensory) have been shown to improve motor function in subjects with PD, including improvement of gait [20–23].

Studies have demonstrated that physiotherapy may improve balance in individuals with PD [24–26]. Researchers suggest that balance training should start before the patient develops a high risk of falls, aiming at prevention of falls through the optimization of compensatory mechanisms [27, 28]. Recently, different exercise modalities have been used in PD patients to promote cognitive engagement, enhanced by feedback (verbal or proprioceptive), attentional demand through cueing, or dual tasking (as in the performing one of two motor activities such as tai chi [29] and tango dancing [30], or the simultaneous performing of motor and cognitive tasks, as in the use of the Nintendo Wii [31]).

Current studies describe how the action mechanisms of external cues are able to promote balance. Evidence shows that an internal model integrates the afferences received by the cortex before the movement execution to the motor-sensory afferences received during or after the execution of the movements. Studies have indicated that cues may minimize a deficit in the generation of the basal ganglia signal, and even more it could function as a guide to a movement previously learned. Therefore, owing to dopamine depletion, PD patients can have difficulties in executing the automatic form of sequential movements. This difficulty could be minimized by the use of external cues [32]. Another possible mechanism would be to increase the attentional engagement on movement [19]. Considering that a deficiency in internal cues affects the automatic execution of movement, attentional strategies will allow a greater attentional control on the task, minimizing deficits in performance [33]. Patients with PD can present difficulty in the activation of balance reactions at the correct time as a main dysfunction; however, the mechanisms of action are unclear [34, 35].

Our hypothesis is that rhythmical cues associated with exercises and motor training may facilitate an anticipatory and compensatory reaction [27, 28], thus improving gait and preventing falls. In fact, there are cognitive components in the generalization and maintenance of balance training that may be applied to the daily activities. The aim of this proposed randomized controlled trial protocol is to evaluate the effects of a motor program based on improving balance associated with rhythmical auditory cues.

## Methods

### Trial design

This was a parallel, prospective, single-blind, randomized clinical trial.

### Design and procedures

**Fig. 1:**
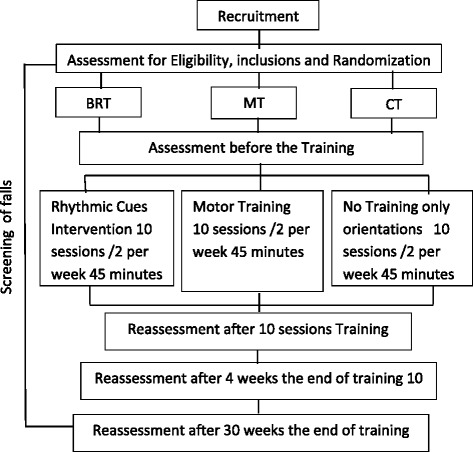
Design of the study. BRT: Balance Rhythmical Training. MT: Motor Training. CT: Control Group

First, we defined the training guidelines and its progression. Subsequently, we developed an exercise program specifically to promote balance (active global exercises and rhythmical auditory cues, generated by a metronome). Subjects were randomly distributed among three groups. The first experimental group will be led by a physiotherapist, it will receive motor skill training with rhythmical auditory cues marked by a metronome (GBRT); the second experimental group will receive the same training without rhythmical cues (MT); and the control group (CT) will receive exercises in general orientation only with a general orientation. To verify retention after the training, subjects will be assessed and reassessed as a follow up 4 and 30 weeks after the end of their training (Fig. 1). The patients will be diagnosed by neurologists in the Movement Disorders Ambulatory Clinic of the University of São Paulo Faculty of Medicine Clinics Hospital (MD HC FMUSP). Physiotherapists will recruit patients on the database of the MD HC FMUSP and Motor-Sensory Learning Laboratory. They will conduct a brief telephone interview based on questions about their personal and medical history and self-perceived balance performance. Then, on a first visit, the subjects will be informed about the procedure and they will sign a consensual form.

This study is ongoing in the stage 1. This study was approved by the Ethics Committee of the University of São Paulo Faculty of Medicine - 1.102.464 and the Clinical trials.gov Identifier is NCT02488265.

### Participants

We will examine 150 PD patients before and after training.

#### Inclusion criteria

All of the subjects will be diagnosed by neurologists from the Movement Disorders Ambulatory Clinic of the University of São Paulo Faculty of Medicine Clinics Hospital, according to the UK Brain Bank criteria [36] and they should be at H&Y classification stage II or III, with a Mini Mental Status Examination (MMSE) [37] and they should have a score of above 24. All patients should also present fall history in the past months. They should have the capacity to ambulate independently indoors without aid.

#### Exclusion criteria

These are the presence of neurological, orthopedic or cardiopulmonary problems, an unstable medication regime, and an inability to understand or adhere to the protocol because of, for example, cognitive, auditory or visual problems. Patients receiving physical therapy training will also be excluded from the training.

### Sample size calculation

The sample size calculation was based on the Mini-Balance Evaluation Systems Test (Mini-BESTest) score. The mean adopted—based on a pilot study of PD patients with similar characteristics to those of the patients in this trial—was 27, and indicated a 3-point difference between motor training and rhythmical auditory cues training. Based on this difference, the sample size calculation revealed that 150 patients (50 per group) would be sufficient for a power greater than 90 % (α = 0.05).

### Randomization and blinding

Randomization is achieved through a computer-generated random-sequence table. The subject’s randomization will be carried out by a researcher who is not involved in enrolling the participants, in assigning them to their groups, or in performing follow-up measurements. This researcher will keep the allocation concealed, and will prepare sealed envelopes, which shall be opened individually only when each participant starts their training. Subjects will be distributed among three groups. Only the researcher responsible for conducting the training will know how the participants are distributed. The researcher responsible for follow-up reviews will be aware of the allocations at any time during the data collection. He/she will understand the data analysis process because the evaluator will also be the same researcher that analyzes the full set of data.

### Outcome measures and test procedure

The primary outcome is balance, which will be assessed by the Berg Balance Scale (BBS) [38], postural stress test (PST) [39], push and release test (PRT) [40] and Mini BESTest (MBESTest) [41]. The secondary outcome is gait, which will be evaluated by the timed up and go test (TUG) [42] and by the freezing of gait, using the Freezing of Gait Questionnaire [43]. Independence in activities of daily living (ADLs), and motor performance will be assessed by the unified Parkinson’s disease rating scale (UPDRS) [44]. Falls and fear of falling will be evaluated during a variety of everyday activities and measured by the Falls Efficacy Scale-International (FES-I) [45].

The number of falls will be registered by the physiotherapist at the hospital and by the patient at home. A weekly follow-up of falling will be conducted.

### Guidelines

The balance rhythmical training (BRT) guidelines are: 1) self-perception (verbal and visual guidance); 2) motor performance (speed, range of motion, trunk mobility, turning, balance exercises); 3) attention strategies (attention sharing between guidance, environment and your own movement; maintenance of attention during all set strategies); and 4) cues (rhythmical auditory cues).

### The program

The exercise program aimed at improving balance is of 5 weeks’ duration with two sessions a week, 45 min per session, and consists of general physiotherapy exercises. Each session will be divided into five warm-up minutes—30 min for the main part and 10 min for the cool down. General exercises will be taught in each part of the therapy [46, 47], as well as balance improvement exercises, which will be defined according to the program guidelines. The training sessions will be in groups comprising 10 participants and 2 physiotherapists. The physical therapists will place themselves behind the patients so that they can catch or hold the patients if they fall.

### Interventions

#### Training progression

The training progresses and intensifies each week. In the first week, before training, each exercise will be explained and demonstrated by the physiotherapist; patients will be encouraged to pay particular attention to the most difficult aspects of movement execution. The subjects will perform five repeated movements (5 RM) of each exercise.

The session will be divided into three parts:

1. **Warm-up**: Of 5 min’ duration, starting with muscle stretches, and followed by joint movements, global movement of the upper and lower limbs, and a wide range of joint movements, being performed in all axes of movement as well as free active movement of the scapulohumeral joint, wrist and elbows, hip, knees and ankles. The movements will involve flexion and extension, lateral inclination and rotation of the trunk; and flexion and extension, lateral inclination and rotation of the cervical spine. The exercises will be performed with the feet in neutral position, together and apart, with decreasing and increasing of the base of support, with and without support (support bar or chair).
2. **Motor training**: Balance exercises will take 30 min’ duration. They consist of three sections:
  A. Balance—axial and proximal movement displacements in different planes and axes; coordinated movements with upper and lower range and speed; functional reach, weight shifts in different directions (anterior, posterior and lateral), using foam, using stable and unstable ground, mats and disks with textures. Postural reactions, trunk rotation. Head movement: bending, rotation, and leaning sideways (eyes opened and closed).
  B. Gait training: stationary and gait training on a stable and instable surface. In this part, foam of different sizes and densities is used.
  C. Functional movements (stand up from the chair, turn around, and bend over to pick up different objects on the floor—of differing weight, size, texture and color).
3. **Cool down**: Of 10 min’ duration. The execution rhythm becomes progressively slower. Slow walking, breathing exercises associated with free active movement of the upper limbs, global muscular relaxation and stretching. Posture training in the orthostatic position. The exercises will be initially performed in neutral position and the eyes will be opened and closed. The patients will be encouraged to pay attention to the position of the body and limbs in all parts; range of motion; posture; basis of support.

In the first phase the patients may make some mistakes and may need specific instructions on how to correct the movement that they will be attempting to execute (attentional strategies). The exercises will be repeated in subsequent sessions to consolidate learning. The subjects must be capable of associating the corrections to the exercises that were proposed in the first week. In each group repetition, for both groups, the physiotherapist will use a verbal command before each movement change, anticipating it and encouraging the patients to pay particular attention to the most difficult ones. Only the GBRT will be oriented to focus on the rhythmical auditory cues provided by a metronome; in warm-up, the metronome will be set at 100 BPM; in motor training, the metronome will be set at 110–150 BPM, and on cool down the metronome will be set at 80–90 BPM.

In the second week, the subjects will perform a series of 10 repetitions (10 RM) and in the third week, two series of 10 repetitions (20 RM). In the fourth week, the subjects must be able to execute 20 RM of the exercise sequences with increasing speed (Additional file 1: Table S1).

### Weekly screening of progression and falls

Scheduled screening of progression in training will be used to indicate the capacity to continue to progress. The patients, who complete all the settings with good and high quality performance in 10 RM, will be able to progress to the next week. Those patients who will not show a good quality of performance will receive additional guidance on the exercises and will receive special attention from the orientation physiotherapist in terms of how to perform the exercises correctly. Thereby, if these patients complete all the parts with good and high quality of performance in 10 RM, they will be able to progress to the next week (Additional file 2: Table S2 and Additional file 3: Table S3).

### Screening of progression and falls

The number of falls will be noted by the physiotherapist at the hospital and by the patient at home. Weekly questionnaires will be used to follow up on any falls.

### Statistical analysis

Baseline values of the demographic characteristics and primary and secondary outcome measures of the participants in the MT, CG and the BRT will be compared using an unpaired *t*-test.

The Lilliefors and Levene tests will be used to examine the normality and homogeneity of variance for the primary and secondary outcome measures.

The participants’ performance in the training will be analyzed by three repeated-measures analyses of variance (RM-ANOVA), one for each part of the training (i.e., part 1, part 2 and part 3, with the training session as the within-group factor.

The training effects for each primary and secondary outcome measure will be analyzed for the three training conditions (i.e., control, motor and experimental) at the four assessment time points (i.e., Before the Training (BT), After the Training (AT), Follow up 1 and Follow up 2) using a mixed-design ANOVA with training as the between-group factor and the assessment time point as the within-group factor.

The effect sizes (ES) will be calculated for all comparisons at alpha = 0.05. A Tukey HSD post-hoc test will be used for multiple comparisons. The statistical software Statistica 11 from StatSoft (United States of America (USA)) will be used for all analyses, and p-values below 5 % will be considered to be statistically significant.

## Discussion

Even with the ideal medical treatment, PD patients still present problems with functional activities, gait and balance [2]. To minimize these deficits, physiotherapy is prescribed as a coadjuvant to drug treatment, being more effective when integrated with specific training linked to sensorial cues and cognitive strategies [16–18, 20, 21].

Physiotherapy exercises lead to improved performance in PD patients, as shown in the literature [22, 28].

As physiotherapy has a highly beneficial effect on balance [24], there is some distinction among compensatory mechanisms that could be useful in gait and balance therapeutic intervention. Therefore, our hypothesis is that rhythmical cues associated with exercises and motor training can facilitate an anticipatory and compensatory reaction [27, 28], improving gait and preventing falls, in the following ways: 1) by increasing attentional engagement, thus promoting an improvement in performance [19]; 2) by increasing sensory-motor integration through thalamic activation of subcortical structures from the internal globus pallidus and cerebellum [32]; and 3) by stimulating internal signals from the basal nucleus, facilitating previously learned movements [34].

The exercises and motor training proposed in this study may improve the performance of balance-related activities in PD. The long-term effects of the exercises and motor training in this population are not clear [25]; however, the precocious and regular physiotherapeutic intervention in PD preserves and improves motor function [15], which suggests a neuroprotective effect [19]. The disease is progressive and the motor training and exercises should be adapted to the patient’s needs and PD progression [16].

It is likely that PD patients diagnosed by neurologists in the Movement Disorders Ambulatory Clinic of the University of São Paulo Faculty of Medicine Clinics Hospital, H&Y stage II–III, will be able to learn how to use the compensatory mechanisms necessary to improve postural instability. The inclusion of rhythmical auditory cues in the motor program may help to improve balance in this group of PD patients [26]. We intend to promote strong evidences that this training program may be more effective than others and will be save compensatory and anticipatory reactions at the exact moment that a response is necessary to prevent a fall. Application and progression of the motor program may promote improvement in automatic postural responses. Such responses are influenced by cortical processing related to learning, previous experiences and initial postural conditions [5].

The cues mostly influenced temporal aspects, facilitating sensory-motor integration, as suggested by previous studies [19]. Therefore, auditory cues may facilitate anticipatory responses sufficiently early to avoid loss of balance in the same way that they may promote compensatory responses. The compensatory adjustments depend on detection of the imbalance through different sensorial modalities [6–9]. A possible explanation for this mechanism of action is that, under challenging conditions, PD individuals prioritize voluntary tasks over postural control [11]. In addition, rhythmical cues may increase attentional control, serving as dual-task training.

We suggest that BRT may facilitate attentional engagement with the tasks. External cue stimulation of the sensorial systems may be a good strategy for improving sensorial integration and compensating for basal nucleus deficits. One explanation for the transfer of the benefits of exercise may pertain to the repair or consolidation of overlapping motor circuitry involved in cognitive and automatic components of movements [19]. In fact, there are many cognitive aspects that may assist with the generalization and maintenance of BRT, which can then be applied to activities of daily living.

We suggest that balance training should be started early, as falls occur clinically when the patient has already exhausted all compensatory resources used by the nervous system, which is compromised by basal ganglia deficits, and when postural and cognitive responses are no longer effective [27, 28]. The long-term follow up in the proposed study will be very important to verify important questions concerning the potential effects of, and limitations of, balance training. Our belief is that this trial will provide evidences about the effectiveness of rhythmical cues on balance in PD patients, providing a therapeutic strategy that can be used to treat such dysfunctions.

The physiotherapy proposed to improve balance in individuals with PD is effective, low-cost, and easy to apply. This study contributes to the subject knowledge, with specific knowledge about balance in PD patients and the mechanisms involved in motor control. It also provides data about functional diagnosis, prognosis, clinical programs, and the management of balance treatment programs. This program may also be allied to different sorts of parkinsonisms and other motor disorders that influence balance.

## Additional files

Additional file 1: Table S1. Interventions. (PDF 246 kb) Additional file 2: Table S2. Weekly Schedule Screening of Progression. (PDF 344 kb) Additional file 3: Table S3. Weekly Screening of falls. N: Number of Falls. (PDF 376 kb)

## Abbreviations

PD: Parkinson’s disease
BRT: Balance training with rhythmical cues
MT: Motor training
CG: Control group
RAC: Rhythmic auditory cues
RM: Repeated movements
BBS: Berg Balance Scale
PST: Postural stress test
PRT: Push and release test
MBESTest: Mini-Balance Evaluation Systems test
TUG: Timed up and go test
ADLs: Activities of daily living
UPDRS: Unified Parkinson’s disease rating scale
GBRT: Motor training with rhythmical cues group
