# Supplementary material for: Randomized controlled trial protocol: balance training with rhythmical cues to improve and maintain balance control in Parkinson’s disease
Source: BMC Neurol. 2015 Sep 7;15:162. doi: 10.1186/s12883-015-0418-x (PMC4561447; doi:10.1186/s12883-015-0418-x)
Supplement: Additional file 1: Table S1. — Interventions. (PDF 246 kb) [file 12883_2015_418_MOESM1_ESM.pdf]

Parts of the Training	Interventions	Orientations
Warm-up<br>5 minutes	Starting by muscle stretching, joint movements, global movement of upper and lower limbs, and joint movements of wide range, being performed in all axes of movement as well as free active movement of the scapulohumeral joint, wrist and elbows, hip, knees and ankles. Flexion and extension, lateral inclination and rotation of the trunk; flexion and extension, lateral inclination and rotation of the cervical. Only GBRT – cues with metronome- 100 BPM.	The exercises were performed with the feet on neutral position, together and apart, with decreasing and increasing of the base of support and with and without support (support bar or chair)
Motor Training<br>30 minutes	Consisted on 3 sets :<br>A. Balance - Axial and proximal movements displacements in different planes and axis; coordinated movements with Upper and Lower range and speed; Functional reach, weight shifts in different directions (anterior, posterior and lateral), using foam, using stable and unstable ground, mats and disks with textures. Postural reactions, trunk rotation. Head movement: bending, rotation, and leaning sideways (eyes open and close).<br>B. Gait training: stationary and gait training on stable and instable surface. In this part we used foam in different sizes and densities.<br>C. Functional movements (stand up from the chair, turn around, and bend over to pick up different objects on the floor- weight, size, texture and color).<br>Only GBRT – cues with metronome- 110 to 150 BPM.	The exercises were performed with the basis in neutral position and eyes opened and closed.<br>Pay attention in all sets in body and limbs position; range of motion; posture; basis of support.
Cool down<br>10 minutes	The execution rhythm becomes progressively slower. Slow walking, breathing exercises associated to free active movement of upper limbs, global muscular relaxation and stretching. Posture training in up orthostatic position. Only GBRT – cues with metronome 80 to 90 BPM	

Additional file 1: Table S1- Interventions
