# Supplementary material for: Randomized controlled trial protocol: balance training with rhythmical cues to improve and maintain balance control in Parkinson’s disease
Source: BMC Neurol. 2015 Sep 7;15:162. doi: 10.1186/s12883-015-0418-x (PMC4561447; doi:10.1186/s12883-015-0418-x)
Supplement: Additional file 2: Table S2. — Weekly Schedule Screening of Progression. (PDF 344 kb) [file 12883_2015_418_MOESM2_ESM.pdf]

Week<br>1, 2 ,3 ,4 e 5	Complete<br>______RM	Performance<br>Quality of movements	Able to continue progression
**Activities Part 1**	○ YES<br>○ NO<br>______RM	○ Poor<br>○ Good<br>○ High Quality	○ YES<br>○ NO___________________________________<br>____
**Activities Part 2 Set 1**	○ YES<br>○ NO<br>______RM	○ Poor<br>○ Good<br>○ High Quality	○ YES<br>○ NO___________________________________<br>____
**Activities Part 2 Set 2**	○ YES<br>○ NO<br>______RM	○ Poor<br>○ Good<br>○ High Quality	○ YES<br>○ NO__________________________________<br>____
**Activities Part 2 Set 3**	○ YES<br>○ NO<br>______RM	○ Poor<br>○ Good<br>○ High Quality	○ YES<br>○ NO__________________________________<br>____
**Activities Part 3**	○ YES<br>○ NO<br>______RM	○ Poor<br>○ Good<br>○ High Quality	○ YES<br>○ NO__________________________________<br>____

Additional file 2: Table S2 - Weekly Schedule Screening of Progression
