# Supplementary material for: Randomized controlled trial protocol: balance training with rhythmical cues to improve and maintain balance control in Parkinson’s disease
Source: BMC Neurol. 2015 Sep 7;15:162. doi: 10.1186/s12883-015-0418-x (PMC4561447; doi:10.1186/s12883-015-0418-x)
Supplement: Additional file 3: Table S3. — Weekly Screening of falls. N: Number of Falls. (PDF 376 kb) [file 12883_2015_418_MOESM3_ESM.pdf]

Week	Number of Falls	Time	Place / Environment/Circumstance	Medication	FREEZING
**1**	○ YES<br>○ NO<br>N________	○ Morning ( )<br>○ Afternoon ( )<br>○ Night ( )	Dual task: ( ) YES ( )NO<br>___________________________________<br>___________________________________	○ On<br>○ Off	○ YES<br>○ NO
**2**	○ YES<br>○ NO<br>N________	○ Morning ( )<br>○ Afternoon ( )<br>○ Night ( )	Dual task: ( ) YES ( )NO<br>___________________________________<br>___________________________________	○ On<br>○ Off	○ YES<br>○ NO
**3**	○ YES<br>○ NO<br>N________	○ Morning ( )<br>○ Afternoon ( )<br>○ Night ( )	Dual task: ( ) YES ( )NO<br>___________________________________<br>___________________________________	○ On<br>○ Off	○ YES<br>○ NO
**4**	○ YES<br>○ NO<br>N________	○ Morning ( )<br>○ Afternoon ( )<br>○ Night ( )	Dual task: ( ) YES ( )NO<br>___________________________________<br>___________________________________	○ On<br>○ Off	○ YES<br>○ NO
**5**	○ YES<br>○ NO<br>N________	○ Morning ( )<br>○ Afternoon ( )<br>○ Night ( )	Dual task: ( ) YES ( )NO<br>___________________________________<br>___________________________________	○ On<br>○ Off	○ YES<br>○ NO

Additional file 3: Table S3 – Weekly Screening of falls. N: Number of Falls.
